# An overview of neuro-ophthalmic disorders at Jenna Ophthalmic Center, Baghdad, Iraq (2021-2022)

**DOI:** 10.25122/jml-2023-0499

**Published:** 2024-01

**Authors:** Husam Abdulhadi Majeed, Yasser Al-Rubiay, Ali Abdulkareem Abbas, Mohamed Esam AL Nuaimi, Hayder Mohammed Khammas, Zaid Abdulkhalik Alsaedi, Aows Maan Al Jammal, Mohamed Mosa Abdlhasn, Ali Mhawi Abdul-Gaffar, Omar Saleem Mohammed, Zainab Basim Abbood, Badr Daham Badr, Zainab Kadhum Fahad, Hayder Abd-alkhaliq Abd-alzahra, Hasan Sameer Al-dabbag, Labeeb Mahmood, Maryam Fawzi Talib Al-Qaseer, Zainab Nadom Hamoodi Al-Khafaji, Laith Shareef

**Affiliations:** 1 Ibn Al-Haitham Teaching Eye Hospital, Baghdad Al-Russafa Health Directorate, Baghdad, Iraq; 2 Department of Surgery, College of Medicine, University of Baghdad, Baghdad, Iraq; 3 Al-Kindy College of Medicine, University of Baghdad, Baghdad, Iraq; 4 Department of Ophthalmology, Ghazi Al Hariri Teaching Hospital, Baghdad, Iraq; 5 Department of Ophthalmology, Basra Teaching Hospital, Basra, Iraq; 6 Department of Surgery, Nineveh College of Medicine, Nineveh, Iraq; 7 Department of Ophthalmology, Azadi Teaching Hospital, Duhok, Iraq; 8 Imamain Al-Kathimain Medical City, Al-Karkh Health Directorate, Baghdad, Iraq; 9 Diwaniya Teaching Hospital, Al Diwaniyah, Iraq; 10 Al-Habobi Teaching Hospital, Nasiriyah, Iraq; 11 Al-Najaf Teaching Hospital, Al-Najaf, Iraq; 12 Al-Shaheed Al-Sadir Hospital, Baghdad Al-Russafa Health Directorate, Baghdad, Iraq; 13 Department of Surgery, College of Medicine, Mustansiriyah University, Baghdad, Iraq; 14 Department of Pharmacy, Al-Rasheed University College, Baghdad, Iraq

**Keywords:** neuro-ophthalmology, disorders, blindness, ischemic optic neuropathy, ocular morbidity

## Abstract

Neuro-ophthalmic disorders are often documented individually for each illness, with little data available on their overall incidence and pattern. The overall incidence of neuro-ophthalmic illnesses in Iraq is still not recorded. This study aimed to evaluate the clinical, demographic, and etiological features of patients seeking consultation at an Iraqi neuro-ophthalmology clinic. A prospective cross-sectional observational research was conducted at the Janna Ophthalmic Center in Baghdad, Iraq. The center serves a diverse patient population from various governorates. All newly diagnosed patients with neuro-ophthalmic disorders who visited the neuro-ophthalmological clinic, regardless of gender or age group, were included. The neuro-ophthalmologist established a diagnosis for each case by reviewing the patient's medical history, doing physical examinations, administering specific tests, and, in certain cases, using neuroimaging methods. The duration of the study was extended from March 2021 to November 2022. Among the 6440 patients evaluated, 613 cases were confirmed at the neuro-ophthalmology clinic. Ischemic optic neuropathy (NAION, AION, and PION) was the most prevalent diagnosis, accounting for 17.61% of newly reported cases in the field of neuro-ophthalmology. This was followed by sixth nerve palsy. Diabetes mellitus affected 42.7% of the cases, followed by hypertension, which affected 39.3% of the participants. The incidence of neuro-ophthalmic diseases tended to be high. Ischemic optic neuropathy and sixth nerve palsy, traumatic/compressive optic neuropathy, and papilledema were the most common neuro-ophthalmic disorders reported.

## INTRODUCTION

The field of neuro-ophthalmology emerged as a recognized medical specialization in the 1960s and has shown significant growth in subsequent years [1]. Neuro-ophthalmology combines the disciplines of neuroscience and ophthalmology, focusing on studying disorders of the neurological system manifesting as visual dysfunction [2]. The visual pathways, which connect the retina to the visual cortex, and the oculomotor system, which links the eye muscles to the cortical centers, establish direct connections with a significant portion of the central nervous system. By evaluating these connections, neuro-ophthalmologists can make assumptions about the severity and specific location of impairments [3]. Patients may exhibit various ocular manifestations, including diminished visual acuity, temporary visual impairment, double vision, atypical eye movements, abnormalities in eyelid function, irregularities in pupil size, and sometimes, perceptual distortions [4,5].

Neuro-ophthalmic diseases are not very common, but they can have serious consequences and even be life-threatening [6]. These diseases have a significant role in the development of ocular morbidity [7]. Disorders affecting the optic nerve are often seen as contributing factors to the onset of blindness [8,9]. These disorders can include a range of conditions, such as optic neuritis and atrophy resulting from diverse causes, papilledema, optic nerve malignancies, and other heterogeneous neuropathies [10]. Proptosis may also be caused by malignancies affecting the optic nerve and meninges [11,12]. Ocular motor nerve palsies are significant etiological factors contributing to the development of strabismus (commonly known as squint) and diplopia (double vision) [13].

Neuro-ophthalmic diseases primarily affect two aspects of vision: (1) the afferent visual system, resulting in different types of visual dysfunction, and (2) the efferent path, resulting in central ocular-motor illnesses, ocular-motor cranial neuropathies, gaze instabilities, and pupillary illnesses [14]. These conditions can also impact systemic functions related to the neuro-muscular junction or the muscles exterior to the eye [15]. Changes in the sensory and motor pathways may arise from many circumstances, such as autoimmune disorders, infectious, inflammation, ischemic, traumatizing, compressive, inherited, or degenerative diseases [16]. It is not uncommon for a neuro-ophthalmic dysfunction, such as inflammatory optic neuropathy, to serve as an early indication of an underlying neurological disorder, such as multiple sclerosis [17]. Similarly, the optic nerve head (ONH) swelling may serve as the only indication of heightened intracranial pressure resulting from critical brain diseases that need immediate medical attention [18,19].

Studies have been conducted in different countries to determine the incidence of specific neuro-ophthalmic diseases. For example, a study conducted in the United States found that the incidence rate of non-arteritic anterior ischemic optic neuropathy (NAION) among individuals aged 50 years and older was 10 per 100,000 in Olmsted County, Minnesota [20]. Another study documented the yearly incidence rates of arteritic and nonarteritic anterior ischemic optic neuropathy (AION) as 0.36 and 2.30 per 100,000 individuals, respectively. These findings were specifically seen among patients 50 years of age or older [21]. Optic neuropathies were the most frequent cause of neuro-ophthalmic disorders in a study conducted in France [22].

The incidence of optic neuritis varies in different countries, with reported rates ranging from 1.03 per 100,000 in Japan, 1.46 per 100,000 in Sweden, and 1.60 per 100,000 in Croatia [23, 24]. However, there is limited research on the epidemiology of less prevalent disorders, such as Leber's optic neuropathy [25]. Additionally, there is a lack of available data on the incidence of neuro-ophthalmic diseases in the Middle Eastern population and other Asian populations. Neuro-ophthalmic disorders are often documented individually for each illness, with little comprehensive data available on their overall incidence and pattern. The overall incidence of neuro-ophthalmic illnesses in Iraq is still not recorded. This research aimed to assess the clinical, demographic, and etiological characteristics of patients seeking consultation at a neuro-ophthalmology clinic in Iraq over one year.

## MATERIAL AND METHODS

### Study design and setting

This study used a prospective cross-sectional observational methodology to examine the incidence of neuro-ophthalmic disorders in Iraq. The present investigation adhered to the STROBE guidelines for reporting cross-sectional observational research and the principles outlined in the Declaration of Helsinki for biomedical research [26,27]. The study was conducted at a single center, Janna Ophthalmic Center, based in Baghdad, Iraq. The facility serves a diverse patient population from multiple governorates. The patient recruitment method included people who were attending the facility for routine follow-up appointments as well as those seeking medical counseling for ocular conditions. The selection of this facility was based on its attributes as being a hospital with a wide range of subspecialties, staffed by qualified medical professionals, equipped with advanced diagnostic tools, and an abundant patient load that closely reflects the general community. The study was conducted between March 2021 and November 2022.

### Inclusion criteria

All newly diagnosed patients with neuro-ophthalmic illnesses regardless of gender or age group, who attended the neuroophthalmological clinic were included.

### Exclusion criteria

Patients who missed scheduled appointments were excluded from the study as their absence could have impacted the accuracy and completeness of the data collected. Patients with psychological illnesses that may have affected their ability to provide reliable data were also excluded to ensure the validity of the study. Similarly, patients who were illiterate or had significant difficulty reading were excluded, as reading ability was crucial for understanding and completing data collection materials. Additionally, patients who were unable or refused to provide informed consent were excluded to uphold ethical considerations. Lastly, patients who refused to participate in the data collection process were also excluded, as their unwillingness to participate could have hindered the collection of necessary data.

### Patient assessment and data collection

The initial manifestation of symptoms was determined based on self-reporting from patients. To establish a definitive diagnosis, the neuro-ophthalmologist performed a comprehensive evaluation, including a detailed medical history, physical examination, specific tests, and sometimes neuroimaging. The demographic characteristics, including age and gender, and the primary symptoms and duration, were documented. The primary clinical manifestations were documented in each instance. The measurement of distant visual acuity was conducted using Snellen's chart. In cases where the visual acuity was below 6/60, the individual's capacity to recognize finger counting, detect hand movement, or detect light was assessed. The external ocular examination was conducted with the pen-torch and the slit-lamp biomicroscope. A summary of devices and equipment used in the study is listed in Table 1. The evaluation of optic nerve lesions included color desaturation tests and visual field assessment. The spectrum of neuro-ophthalmic illnesses examined in this research included (1) central nystagmus, (2) congenital optic anomalies (myelinated nerve fiber layer [NFL], disc coloboma, disc hypoplasia), (3) cortical pathology (cerebrovascular accident [CVA], tumors, infection), (4) fourth nerve palsy, (5) functional visual loss, (6) headache syndromes (migraine, cluster, trigeminal neuralgia), (7) Leber hereditary optic neuropathy (Kjer Behr LHON), (8) ischemic optic neuropathy (NAION, AION, posterior ischemic optic neuropathy [PION] [28]), (9) miscellaneous, (10) multiple sclerosis (optic neuritis, internuclear ophthalmoplegia [INO], wall-eyed bilateral internuclear ophthalmoplegia [WEBINO]), (11) multiple nerve palsies (ophthalmoplegia), (12) myopathies (myasthenia, chronic progressive external ophthalmoplegia [CPEO], Botox), (13) Optic neuritis (not associated with multiple sclerosis), (14) papilledema, (15) pupil anomalies (Adies, traumatic, Horner), (16) seventh nerve palsy (acute only), (17) sixth nerve palsy, (18) third nerve palsy, (19) traumatic/compressive optic neuropathy.

**Table 1:** A list of the devices used during the examination

N	Device name	Model	Serial number	Manufacturing date
1	Slit lamp	700GL	0715376	2015
2	Auto refractokeratometer	ARK-1	433133	2016
3	Auto tonometer IOP	NT-530P	334331	2021
4	Visual field machine	KOWOA AP-7000	30000201157	2018
5	OCT machine	HOCT-1F	1CT00F19D009	2019

Auto tonometer IOP, Automated Tonometer for Intraocular Pressure; OCT machine, Optical Coherence Tomography machine.

### Bias

The study participants frequently underwent an initial evaluation with a general ophthalmologist before being referred to the neuro-ophthalmology clinics. This approach was implemented to ensure that the study sample primarily consisted of individuals likely to receive an accurate diagnosis of a neuro-ophthalmic disorder. Additionally, this step aimed to minimize the potential bias arising from misclassification.

### Statistical analysis

The statistical analyses were conducted using IBM SPSS Statistics for Windows (RRID: SCR_002865), version 23. The graphical illustrations were generated using GraphPad Prism 8 for Windows. The continuous and categorical data distributions were reported using the mean and standard deviation for continuous variables, and frequency and percentages for categorical variables. A Chi-square test was conducted to examine the relationship between demographic data and neuro-ophthalmic conditions. A *P* value less than 0.05 was considered statistically significant.

## RESULTS

**Figure 1:**
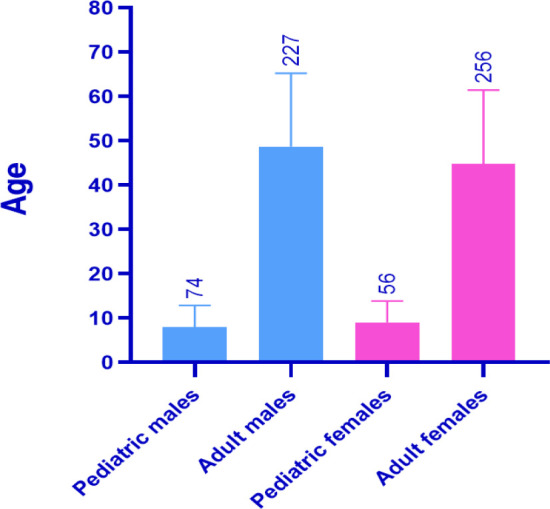
Gender distribution in the pediatric vs. adult population

Over the 1.5-year study period, 6440 patients were referred to various specialist clinics at our institution. Of these, 613 cases were confirmed through consultation with the neuro-ophthalmology clinic, resulting in an incidence rate of 9.51%. The average age of participants was 38.52 ± 21.64 years, ranging from 1 to 88 years. Gender was evenly distributed, with 59.1% female and 49.1% male participants. Most of the patients (64.3%) were over the age of 30. The group also included pediatric patients, comprising 22.8% of the total population, as presented in Table 2 and Figure 1.

**Table 2:** Demographic characteristics

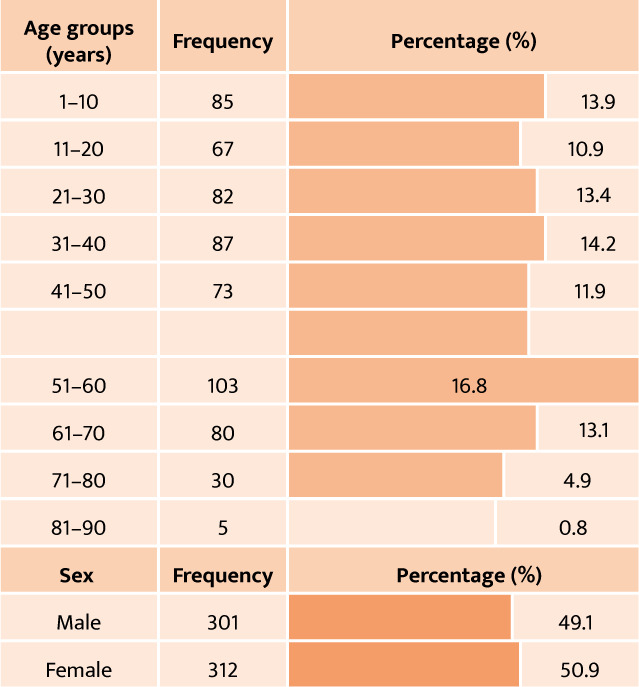

Ischemic optic neuropathy (NAION, AION, PION) emerged as the prevailing diagnosis, accounting for 17.61% of newly reported cases in the field of neuro-ophthalmology. Following this, the prevalence rates of sixth nerve palsy, traumatic/compressive optic neuropathy, and papilledema were 10.92%, 8.8%, and 8.64%, respectively. This study revealed that traumatic/compressive optic neuropathy was the prevailing neuro-ophthalmic illness in individuals below the age of 50, whereas ischemic optic neuropathy (specifically NAION, AION, and PION) was the predominant condition among patients aged 50 and older, as seen in Table 3.

**Table 3:** Types of neuro-ophthalmic diseases

Diagnosis	Age groups		Gender		Total	(%)	*P* value
	< 50 y	≥ 50 y	M	F			
Central nystagmus	14	1	7	8	15	2.44	*P* > 0.05
Congenital optic anomalies (myelinated NFL, disc coloboma, disc hypoplasia)	19	2	6	15	21	3.43	*P* > 0.05
Cortical pathology (CVA, tumors, infection)	34	27	29	32	61	9.95	*P*> 0.05
Fourth nerve palsy	14	6	15	5	20	3.26	*P* < 0.05
Functional visual loss	13	2	4	11	15	2.44	*P* > 0.05
Headache syndromes (migraine, cluster, trigeminal neuralgia)	20	7	11	16	27	4.40	*P* > 0.05
Hereditary optic neuropathy (Kjer Behr LHON)	18	1	14	5	19	3.09	*P* < 0.05
Ischemic optic neuropathy (NAION AION PION)	23	85	58	50	108	17.61	*P* > 0.05
Miscellaneous	29	9	18	20	38	6.19	*P* > 0.05
Multiple sclerosis (optic neuritis, INO, WEBINO)	24	1	7	18	25	4.07	*P* < 0.05
Multiple nerve palsies (ophthalmoplegia)	3	5	6	2	8	1.30	*P* > 0.05
Myopathies (myasthenia, CPEO, MD, Botox)	18	6	7	17	24	3.91	*P* < 0.05
Optic neuritis (NOT to MS)	17	0	7	10	17	2.77	*P* > 0.05
Papilledema	47	6	15	38	53	8.64	*P* < 0.05
Pupil anomalies (Adies, traumatic, Horner)	7	0	3	4	7	1.14	*P* > 0.05
Seventh nerve palsy (acute only)	4	3	5	2	7	1.14	*P* > 0.05
Sixth nerve palsy	29	38	41	26	67	10.92	*P* < 0.05
Third nerve palsy	7	20	17	10	27	4.40	*P* > 0.05
Traumatic/compressive optic neuropathy	43	11	31	23	54	8.80	*P* > 0.05

NFL, Myelinated Nerve Fiber Layer; CVA, Cerebrovascular Accident; LHON, Leber's Hereditary Optic Neuropathy; NAION, Non-Arteritic Anterior Ischemic Optic Neuropathy; AION, Arteritic Anterior Ischemic Optic Neuropathy; PION, Posterior Ischemic Optic Neuropathy; INO, Internuclear Ophthalmoplegia; WEBINO, Wall-Eyed Bilateral Internuclear Ophthalmoplegia; CPEO, Chronic Progressive External Ophthalmoplegia; MD, Mitochondrial Myopathy; MS, Multiple Sclerosis.

**Figure 2:**
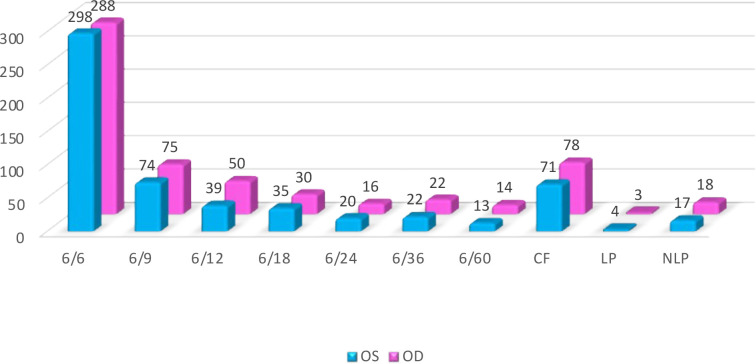
Comparative analysis of BCVA between right (OD) and left (OS) eyes

A significant proportion of participants, including 48.6% in the left eye (OS) and 47% in the right eye (OD), had a visual acuity of 6/6, which indicates their ability to see details from a distance of 6 meters (20 feet) in a manner equivalent to those with normal vision. 12.1% of the patients had a visual acuity of 6/9 in the OS and 12.2% in the OD. The rest of the findings are presented in Table 3 and Figure 2. A total of 11.6% of the OS evaluated showed an ability to count fingers at a given distance. Similarly, 12.7% of the OD had the ability to count fingers at the same distance. This testing procedure is only used after establishing the patient's inability to recognize letters on the visual acuity chart. A total of 0.7% of the OS and 0.5% of the OD could perceive light. This particular testing approach is limited to cases when patients show little or insignificant progress in the Hand Motion test. During this examination, the examiner utilizes a penlight to illuminate the patient's pupil. Subsequently, the patient is instructed to either indicate the location of the light source or articulate the specific direction from which the light originates. In cases when the patient exhibits a complete absence of light perception, it is accepted to document this condition using the acronym NLP, which stands for no light perception. An individual who cannot see the light in one eye is classified as blind in that particular eye. When NLP is seen in both eyes, the patient is clinically diagnosed with complete blindness. The reported percentages of NLP in the left eye (OS) and right eye (OD) were 2.8% and 2.9%, respectively (Table 4 and Figure 2).

**Table 4:** Differences in best corrected visual acuity measurements in the OS vs. OD

	OS BCVA		OD BCVA		*P* value
	Frequency	Percent	Frequency	Percent	
6/6	298	48.6	288	47.0	*P* > 0.05
6/9	74	12.1	75	12.2	*P* > 0.05
6/12	39	6.4	50	8.2	*P* > 0.05
6/18	35	5.7	30	4.9	*P* > 0.05
6/24	20	3.3	16	2.6	*P* > 0.05
6/36	22	3.6	22	3.6	*P* > 0.05
6/60	13	2.1	14	2.3	*P* > 0.05
CF	71	11.6	78	12.7	*P* > 0.05
LP	4	0.7	3	0.5	*P* > 0.05
NLP	17	2.8	18	2.9	*P* > 0.05

CF, counting fingers; LP, light perception; NLP, No light Perception; OD oculus dexter (the right eye); OS oculus sinister (the left eye); BCVA, Best Corrected Visual Acuity.

**Figure 3:**
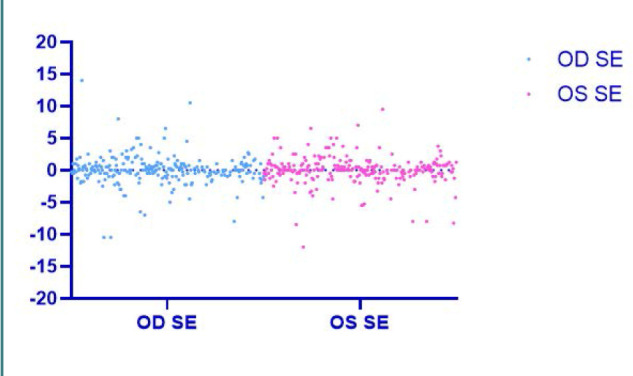
Spherical equivalent measurements in OD vs. OS

There was no statistically significant difference seen in the measurements of the spherical equivalent between the left (OS) and the right eyes (OD) of the patients (*P* = 0.602). The mean value of the spherical equivalent was -0.1403 in the left eyes and -0.1208 in the right eyes, as shown in Table 5 and Figure 3.

**Table 5:** Spherical equivalent measurements in OD vs. OS

Examined eye	Minimum	Maximum	Mean	Std. Deviation	*P* value
OD	-10.50	14.00	-0.1208	2.09	0.602
OS	-12.00	9.50	-0.1403	2.07	

The patterns of eye involvement in patients significantly differed across diagnoses. For conditions like cortical pathology and papilledema, there was a statistically significant tendency for both eyes to be affected rather than just one (*P* < 0.05). On the other hand, multiple sclerosis (optic neuritis, INO, WEBINO) was predominantly detected in the left eye (*P* < 0.05), while fourth nerve palsy was reported significantly on the right eye side, as presented in Table 6.

**Table 6:** Distribution of ocular disorders by affected eye in the study population

Diagnosis	Affected eye			Total	*P* value
	OD	OS	OU		
Central nystagmus	1	2	12	15	*P* > 0.05
Congenital optic anomalies (myelinated NFL, disc coloboma, disc hypoplasia)	7	7	7	21	*P* > 0.05
Cortical pathology (CVA, tumors, infection)	4	11	46	61	*P* < 0.05
Fourth nerve palsy	13	6	1	20	*P* < 0.05
Functional visual loss	0	0	15	15	*P* < 0.05
Headache syndromes (migraine, cluster, trigeminal neuralgia)	7	5	15	27	*P* > 0.05
Hereditary optic neuropathy (Kjer Behr LHON)	0	0	19	19	*P* < 0.05
Ischemic optic neuropathy (NAION AION PION)	48	40	20	108	*P* < 0.05
Miscellaneous	6	6	26	38	*P* < 0.05
Multiple sclerosis (optic neuritis, INO, WEBINO)	7	12	6	25	*P* < 0.05
Multiple nerve palsies (ophthalmoplegia)	5	2	1	8	*P* > 0.05
Myopathies (myasthenia, CPEO, MD, Botox)	2	2	20	24	*P* < 0.05
Optic neuritis (NOT to MS)	2	5	10	17	*P* > 0.05
Papilledema	4	3	46	53	*P* < 0.05
Pupil anomalies (adies, traumatic, horner)	4	2	1	7	*P* > 0.05
Seventh nerve palsy (acute only)	4	3	0	7	*P* < 0.05
Sixth nerve palsy	32	33	2	67	*P* < 0.05
Third nerve palsy	11	16	0	27	*P* < 0.05
Traumatic/compressive optic neuropathy	14	20	20	54	*P* > 0.05

OD oculus dexter (the right eye), OS oculus sinister (the left eye), OU oculus uterque (both eyes)

The development of neuro-ophthalmic disorders was significantly influenced by medical background and patient-related factors. The findings of this study indicate that diabetes mellitus had a notable impact on 42.7% of the cases, followed by hypertension, which affected 39.3% of the individuals who had a medical history of these chronic diseases. The data reported in Table 7 indicates that smoking had a detrimental effect on the development of neuro-ophthalmic diseases in 9% of the patients.

**Table 7:** Relationship between medical history and risk factors with the incidence of neuro-ophthalmic diseases

Medical History Diagnosis	Covid-19	Diabetes	Drugs	Hypertension	Lipid abnormalities	Positive family history	Smoking	Trauma	Total
Traumatic/ compressive optic neuropathy	1	4	0	3	0	0	2	4	14
	7.1%	28.6%	0.0%	21.4%	0.0%	0.0%	14.3%	28.6%	100.0%
Third nerve palsy	0	17	0	8	0	0	2	0	27
	0.0%	63.0%	0.0%	29.6%	0.0%	0.0%	7.4%	0.0%	100.0%
Sixth nerve palsy	1	31	0	23	1	0	3	3	62
	1.6%	50.0%	0.0%	37.1%	1.6%	0.0%	4.8%	4.8%	100.0%
Seventh nerve palsy	0	1	0	2	0	0	0	0	3
	0.0%	33.3%	0.0%	66.7%	0.0%	0.0%	0.0%	0.0%	100.0%
Pupil anomalies	0	0	0	0	0	0	1	0	1
	0.0%	0.0%	0.0%	0.0%	0.0%	0.0%	100.0%	0.0%	100.0%
Papilledema	0	2	0	5	0	0	3	1	11
	0.0%	18.2%	0.0%	45.5%	0.0%	0.0%	27.3%	9.1%	100.0%
Optic neuritis	0	1	0	0	0	0	1	0	2
	0.0%	50.0%	0.0%	0.0%	0.0%	0.0%	50.0%	0.0%	100.0%
Myopathies	0	1	3	2	0	0	0	0	6
	0.0%	16.7%	50.0%	33.3%	0.0%	0.0%	0.0%	0.0%	100.0%
Multiple nerve palsies (ophthalmoplegia)	0	5	0	2	0	0	0	1	8
	0.0%	62.5%	0.0%	25.0%	0.0%	0.0%	0.0%	12.5%	100.0%
Multiple sclerosis	1	0	0	0	0	0	0	0	1
	100.0%	0.0%	0.0%	0.0%	0.0%	0.0%	0.0%	0.0%	100.0%
Miscellaneous	0	4	1	9	0	0	0	0	14
	0.0%	28.6%	7.1%	64.3%	0.0%	0.0%	0.0%	0.0%	100.0%
Ischemic optic neuropathy	1	45	0	48	1	0	10	0	105
	1.0%	42.9%	0.0%	45.7%	1.0%	0.0%	9.5%	0.0%	100.0%
Hereditary optic neuropathy	0	2	0	1	0	2	0	0	5
	0.0%	40.0%	0.0%	20.0%	0.0%	40.0%	0.0%	0.0%	100.0%
Headache syndromes	0	3	0	2	0	0	1	0	6
	0.0%	50.0%	0.0%	33.3%	0.0%	0.0%	16.7%	0.0%	100.0%
Functional visual loss	0	4	0	1	0	0	0	0	5
	0.0%	80.0%	0.0%	20.0%	0.0%	0.0%	0.0%	0.0%	100.0%
Fourth nerve palsy	0	2	0	3	1	0	3	3	12
	0.0%	16.7%	0.0%	25.0%	8.3%	0.0%	25.0%	25.0%	100.0%
Cortical pathology	1	16	1	16	0	0	3	1	38
	2.6%	42.1%	2.6%	42.1%	0.0%	0.0%	7.9%	2.6%	100.0%
Congenital optic anomalies	0	0	0	1	0	0	0	0	1
	0.0%	0.0%	0.0%	100.0%	0.0%	0.0%	0.0%	0.0%	100.0%
Central nystagmus	0	0	1	1	0	0	0	0	2
	0.0%	0.0%	50.0%	50.0%	0.0%	0.0%	0.0%	0.0%	100.0%
Total	5	138	6	127	3	2	29	13	323
	1.5%	42.7%	1.9%	39.3%	0.9%	0.6%	9.0%	4.0%	100.0%

## DISCUSSION

The current research was conducted at the neuro-ophthalmology clinic, which is a division of the Janna Ophthalmology Center. Consequently, all participating patients had eye symptoms or impaired visual functions. The overall incidence of neuro-ophthalmic cases among new patients attending the ophthalmology clinic was 9.51%. The incidence rate was higher than that reported in a Nigerian research by Omoti *et al*., who reported an incidence rate of 4.47% [1]. A possible explanation for this variability is the prevalence of certain neuro-ophthalmic illnesses, which are more often seen in Asian populations compared to other ethnic groups [29,30]. The natural environment, social and cultural, and psychological variables may also have an influence [31]. Due to the expansive definition of neuro-ophthalmic illnesses and variations in the criteria used for evaluation, the frequency of certain disorders, as well as the total occurrence, might differ across different studies. There seems to be a positive correlation between advancing age and a higher incidence of neuro-ophthalmic illnesses. Research conducted in Singapore revealed a strong correlation between the occurrence and those over 40, whereas no association was seen with gender [32,33]. The results presented are consistent with what was determined in the present research.

Although this research did not find any statistically significant differences in relation to gender, it is worth noting that there was a slightly higher frequency identified among women. This disparity in prevalence rates between genders may be attributed to biological factors. For instance, the sex steroid hormone in females has significant effects on endothelial cells, leading to vasodilation and increased blood circulation. These actions play a crucial role in preventing ischemic diseases [34]. However, the observed protective effect diminishes significantly in postmenopausal women, as they have inferior cerebrovascular responses compared to males of the same age [18]. Consequently, the biological alteration seen in women aged 50 to 60 may potentially elevate the likelihood of non-arteritic anterior ischemic optic neuropathy and result in similar or slightly higher prevalence rates when compared to men. Furthermore, sex-specific risk factors may probably contribute to the observed disparity across genders. The male population, for example, has a greater propensity for smoking and alcohol usage, which correlates with an increased susceptibility to peripheral vascular disorders [35]. On the other hand, women tend to exhibit elevated levels of plasma total cholesterol and low-density lipoprotein cholesterol, which leads to an increased susceptibility to atherosclerosis and thromboembolism compared to males [36,37].

Research conducted over two years at a tertiary eye clinic in Nigeria documented 76 newly diagnosed cases of neuro-ophthalmic conditions. This accounts for about 4.47% of all new patients seen throughout the study. The study identified ocular motor palsies as the most prevalent neuro-ophthalmic disorder, accounting for 27.6% of cases. Optic neuropathies were the second most common condition, comprising 22.4% of cases, followed by migraine at 14.5%. The most frequently reported symptoms at presentation were impaired vision, reported by 39.5% of patients, followed by double vision (18.4%) and headache (17.1%) [1].

Lim *et al*. [32] conducted a study in Singapore to determine the yearly incidence of neuro-ophthalmic disorders. The study found an incidence rate of 9.81 per 100,000 individuals. The three most prevalent neuro-ophthalmic diseases identified were abducens nerve palsy, anterior ischemic optic neuropathy, and oculomotor nerve palsy, with incidence rates of 1.27, 1.08, and 0.91 per 100,000 individuals, respectively [32].

The present study identified five increasing neuro-ophthalmic illnesses: ischemic optic neuropathy (including NAION, AION, and PION), sixth nerve palsy, cortical pathology (including CVA, tumors, and infection), traumatic/compressive optic neuropathy, and papilledema. The study of Lim *et al*. [20] in Singapore revealed that abducens nerve palsy had the greatest incidence rate of 1.27 per 100,000 per year. This was followed by NAION with an incidence rate of 1.08 per 100,000, oculomotor nerve palsy with an incidence rate of 0.91 per 100,000, and optic neuritis with an incidence rate of 0.83 per 100,000 [32].

The findings of this research indicate that traumatic/compressive optic neuropathy was the most common neuro-ophthalmic disease seen in individuals under the age of 50. Conversely, ischemic optic neuropathy, namely NAION, AION, and PION, was the most prevalent condition among patients aged 50 and older. This finding is consistent with a study conducted by Nattapong *et al*. in a tertiary hospital in Thailand. They identified the same prevalent disease among individuals aged 50 years and above. However, it contradicts the findings regarding the predominant disorder among individuals below 50 years old, as the study reported optic neuritis as the prevalent disorder in this age group [38].

This research revealed that a significant proportion of patients who exhibited neuro-ophthalmic disorders had a BCVA of 6/6. This finding contradicts a study in Thailand, which showed that 40% of patients experiencing reduced vision were classified as blind in the afflicted eye [38]. The etiology of blindness showed variability. While the incidence of non-arteritic anterior ischemic optic neuropathy was very high, it is worth noting that just one participant in the research mentioned above had complete vision loss [39]. Multiple investigations have shown that NAION often manifests with a Snellen visual acuity superior to 6/60 [40].

The present study revealed that the predominant medical comorbidities and patient-related factors were diabetes, hypertension, and smoking. These findings align with a previous study conducted by Lee *et al*. in South Korea, which reported that neuro-ophthalmic patients commonly presented with underlying diseases such as diabetes, hypertension, hyperlipidemia, stroke, myocardial infarction, sleep apnea, pulmonary embolism, and deep vein thrombosis [41].

One of the notable strengths of this research is its prospective methodology for case collection, which allows for the examination of many aspects of the illnesses across different age groups and both genders. One of the limitations of this study is its single-center design. Another limitation is the interdisciplinary nature of certain neurological conditions, such as brain tumors, stroke that involves visual pathways, myasthenia gravis, hemifacial spasm, and blepharospasm, which require the collaboration of multiple medical specialties. Consequently, there is a risk of excluding possible cases that should be managed by neurologists or neurosurgeons. This limitation could contribute to a potential underreporting of the incidence rates of these conditions within the study parameters.

## CONCLUSION

The primary neuro-ophthalmic conditions identified in this study were ischemic optic neuropathy, sixth nerve palsy, traumatic/compressive optic neuropathy, and papilledema. The prevalence of neuro-ophthalmic illnesses varies since it is reliant upon the specific inclusion criteria used in each research. The incidence of neuro-ophthalmic illnesses is generally high.
